# Thyroid-stimulating hormone, fasting blood glucose and suicidal ideation in Chinese adolescents with major depressive disorder: a cross-sectional study

**DOI:** 10.3389/fpsyt.2026.1892174

**Published:** 2026-07-20

**Authors:** Jun Li, Yinghan Tian, Yu Zhuang, Wenzheng Li, Kai Zhang, Huanzhong Liu

**Affiliations:** 1 Department of Psychiatry, The Fourth Affiliated Hospital of Anhui Medical University, Hefei, Anhui, China; 2 Anhui Psychiatric Center, Anhui Medical University, Hefei, Anhui, China; 3 Department of Psychiatry, Hefei Fourth People’s Hospital, Hefei, Anhui, China; 4 Anhui Provincial Key Laboratory for Brain Bank Construction and Resource Utilization, Hefei, Anhui, China

**Keywords:** adolescents, fasting blood glucose, major depressive disorder, suicidal ideation, thyroid-stimulating hormone

## Abstract

**Background:**

Thyroid function and glycolipid metabolic alterations are often associated with major depressive disorder (MDD), yet their roles in suicide risk among adolescents with MDD remain unclear. This study aimed to investigate thyroid-stimulating hormone (TSH) levels, glycolipid metabolism parameters, and their associations with suicidal ideation (SI) in adolescents with MDD.

**Methods:**

This cross-sectional study was conducted at one general hospital and one psychiatric hospital in Anhui Province, China. Socio-demographic data and laboratory parameters were collected from participants, and the patients’ depressive symptoms and SI severity were assessed using the 24-item Hamilton Depression Rating Scale (HAMD-24) and the Positive and Negative Suicide Ideation Inventory (PANSI), respectively. TSH levels and glycolipid metabolism parameters were also measured.

**Results:**

A total of 146 adolescents with MDD and 70 healthy controls (HCs) were enrolled in this study. Compared with HCs, patients had lower fasting blood glucose (FBG) levels (*P* < 0.001). Logistic regression analyses showed that a worse relationship with family, a higher HAMD-24 total score, and higher TSH and FBG levels were independently associated with concurrent SI in adolescents with MDD (all *P* < 0.05). Furthermore, receiver operating characteristic (ROC) curve analysis showed that the combination of these four factors had a good discriminatory ability for SI, with an area under the curve (AUC) of 0.851.

**Conclusion:**

In this cross-sectional study, TSH and FBG levels were associated with SI in adolescents with MDD. Nevertheless, whether these parameters can serve as clinically useful biomarkers requires further validation in larger prospective studies.

## 1 Introduction

In recent years, the prevalence of major depressive disorder (MDD) has continued to rise, positioning it among the primary contributors to the global burden of disease and disability (1). MDD may occur across the lifespan, with the 15–19 age group experiencing the most rapid increase in incidence and disease burden (2). Among adolescents, MDD not only leads to interpersonal and academic dysfunction, but also increases the risk of suicidality (3–5). Although the etiology of MDD remains unclear, accumulating evidence suggests that it is associated with multiple neurobiological alterations, including monoamine neurotransmitter deficiency, hypothalamic-pituitary-thyroid (HPT) axis dysfunction, and metabolic dysregulation (6, 7).

Suicide represents a major global public health challenge, and ranks among the leading causes of death in adolescents. According to the latest World Health Organization (WHO) report (8), suicide is the fourth leading cause of death among adolescents aged 15–19 years and ranks second among females in this age group. Previous studies have shown that most adolescents who experience suicidal behavior are likely to have one or more psychiatric disorders, with MDD demonstrating the strongest association (9). A recent meta-analysis of 26 studies revealed that patients with MDD have an 8.62-fold higher risk of suicide death than the general population (10). Another large-scale case-control study demonstrated that over 40% of suicide decedents had been diagnosed with MDD within one year before death (11). Suicidal ideation (SI), defined as thinking about, considering, or planning suicide, is recognized as an early stage in the development of suicidal behavior. Studies conducted in China reported that the prevalence of SI was 15.4% among children and adolescents (12), and 68% of adolescents with MDD also presented with SI (13). This highlights the significance of early identification and timely intervention targeting SI in adolescents with MDD.

The factors influencing SI in adolescents are complex and encompass multiple levels, including social, psychological, behavioral, and biological domains. A meta-analysis comprising 62 predictive models demonstrated that female sex, depressive symptoms, bullying, and family relationships were independent influencing factors for suicide risk among adolescents (14). For patients with MDD, some studies indicated that age of onset, recurrent depression, and prior suicidal behavior were also significantly associated with SI (15, 16). Despite advances in the identification of factors associated with SI, the exact biological mechanisms underlying SI remain unclear in patients with MDD. The thyroid gland, the largest endocrine gland in the human body, plays a critical role in regulating metabolism and the development of the nervous system (17). Studies have confirmed that thyroid dysfunction is associated with an increased risk of MDD (18). It has been further demonstrated by recent research that the thyroid gland may be involved in the pathophysiological mechanisms underlying suicidal behavior, through its modulation of brain areas implicated in emotion regulation and cognitive control (19). An early study found that SI was negatively correlated with thyroid-stimulating hormone (TSH) levels in female patients with MDD (20). In contrast, two other cross-sectional studies, conducted separately in adults and adolescents with MDD, found that those with suicidal behavior had significantly elevated TSH levels (21, 22). Currently, the association between TSH levels and SI in adolescents with MDD remains unclear and has not been systematically investigated.

Moreover, dysregulation of glycolipid metabolism is also linked to a higher risk of suicidal behavior among patients with major psychiatric disorders (e.g., MDD, bipolar disorders, and schizophrenia), which has been confirmed in previous studies (23–25). Among adolescents with MDD, two recent studies reported that higher total cholesterol (TC) and triglyceride (TG) levels, as well as lower high-density lipoprotein cholesterol (HDL-C) levels, were associated with an increased risk of SI (26, 27). However, two other studies conducted among adults and adolescents with MDD yielded inconsistent results regarding the association between fasting blood glucose (FBG) levels and SI (28, 29).

Therefore, this study aimed to (1) compare the levels of TSH and glycolipid metabolism parameters between adolescents with MDD and HCs, and (2) explore the factors influencing SI, with a particular focus on examining the associations of TSH and glycolipid metabolism parameters with SI in adolescents with MDD.

## 2 Methods

### 2.1 Study design and participants

This cross-sectional study was conducted at the Fourth Affiliated Hospital of Anhui Medical University and Hefei Fourth People’s Hospital in Anhui Province, China, from October 2020 to March 2022. The inclusion criteria for patients were as follows (1): aged 12 to 18 years (2); diagnosed with MDD based on independent assessment by two senior psychiatrists using the Structured Clinical Interview for DSM-5 (SCID-5), with the final diagnosis confirmed by consensus (3); capable of understanding and completing all assessment measures. The exclusion criteria were as follows (1): comorbidity with any other psychiatric disorder (e.g., schizophrenia or bipolar disorder) (2); a history of severe physical illness (e.g., diabetes, hyperlipidemia, or thyroid disease), neurodevelopmental disorders, or substance use disorders (3); use of lithium, thyroxine, immunomodulators, or other medications that may affect thyroid function within the past three months. Healthy controls (HCs) aged 12 to 18 years were recruited from local communities and schools during the study period. All HCs were screened for current psychiatric symptoms and disorders using the SCID-5 administered by senior psychiatrists, and were required to have no personal or family history of psychiatric disorders. It should be noted that the patients in the present study are a subset of those from our previous study (22) and were drawn from the same recruitment pool of adolescents with MDD. The current study addresses a distinct scientific question by specifically examining the associations of TSH and FBG with concurrent SI, whereas the earlier study focused on suicide attempts. Using G*Power (version 3.1.9.7), we calculated the minimum required sample size as 134, assuming an effect size of 0.50 based on prior studies (30), a two-tailed *α* of 0.05 and a statistical power (1-*β*) of 0.80. The final sample consisted of 146 adolescents with MDD.

This study implemented rigorous clinical safety and ethical protocols. During the assessments, participants’ acute suicide risk was evaluated by psychiatrists using the SCID-5 and psychometric instruments, such as the suicide item of the 24-item Hamilton Depression Rating Scale (HAMD-24). Participants identified as being at high risk of suicide were immediately reported to their guardians, underwent clinical review by their attending psychiatrist, and received emergency clinical interventions, including hospitalization, continuous clinical observation, or treatment plan adjustment. The study protocol was approved by the Medical Ethics Committee of the Fourth Affiliated Hospital of Anhui Medical University (202009-kyxm-04), and written informed consent was obtained from all participants and their legal guardians prior to participation. All study procedures strictly adhered to the principles of the Declaration of Helsinki.

### 2.2 Data collection and measurements

#### 2.2.1 Socio-demographic characteristics

Socio-demographic and clinical data were collected using a pre-designed questionnaire, including age (years), sex (male/female), body mass index (BMI, kg/m^2^), parental marital status (normal/separation, divorce or others), relationship with family (good/fair/poor), age of onset (years), duration of illness (months), and the use of psychiatric medications (antidepressants, mood stabilizers, and atypical antipsychotics). Based on the Defined Daily Dose (DDD) conversion method recommended by the World Health Organization (31), the antidepressant dose per patient was converted to fluoxetine equivalents. History of suicide attempts was assessed using a standardized yes/no question: “Over the past year, have you attempted suicide?” (32). Additionally, information regarding suicide attempts was verified by reviewing patient medical records and conducting interviews with family members.

#### 2.2.2 Clinical measurements

The HAMD-24 was used to assess patients’ depressive symptoms over the past week (33). The total score of the HAMD-24 ranges from 0 to 76, with higher scores indicating more severe depressive symptoms. To avoid the potential confounding effect of HAMD item 3 (suicide), we excluded this item and recalculated the modified HAMD-24 total score as the measure of depression severity in all analyses examining the association between depressive symptoms and SI. The severity of SI over the past two weeks was assessed using the 14-item Positive and Negative Suicide Ideation Inventory (PANSI), with each item rated on a 5-point scale from 1 (never) to 5 (always) (34). The scale comprises two subscales: Positive Suicidal Ideation (PSI) and Negative Suicidal Ideation (NSI). The PANSI total score is the sum of reverse-scored PSI and positively scored NSI, with higher scores indicating more severe SI. As reported in previous studies, patients with a mean item score for PSI of ≤ 3.333 and a mean item score for NSI of ≥ 1.625 were considered to have SI (35, 36). It should be noted that the frequency of SI and all subsequent analyses depend on the validity of this classification approach. The Chinese versions of the HAMD-24 and the PANSI have been widely validated and used in clinical research in China (37, 38). In the present study, the Cronbach’s α coefficients of the HAMD-24 and the PANSI were 0.793 and 0.939 respectively.

#### 2.2.3 Laboratory parameter measurements

Following an overnight fast, blood samples were collected from participants between 06:00 and 07:00. Blood samples were centrifuged, and the resulting serum was labeled and stored at -80 °C. All assays were completed within 30 days after blood collection. All serum sample assays were performed by the clinical laboratory at the Fourth Affiliated Hospital of Anhui Medical University. The testing personnel performed daily instrument calibration and internal quality control, strictly followed standard clinical laboratory operating procedures, and ensured intra-assay coefficients of variation (CVs) of < 5% and inter-assay CVs of < 10%. Serum TSH levels (reference range: 0.35-4.94 μIU/mL) were measured using a cobas e 801 electrochemiluminescence immunoassay analyzer (Roche Diagnostics, Shanghai, China). FBG (reference range: 3.9-6.1 mmol/L), TC, TG, HDL-C, and low-density lipoprotein cholesterol (LDL-C) levels were determined by a Siemens Advia Chemistry XPT automatic biochemical analyzer (Siemens, New York, USA).

### 2.3 Statistical analysis

Statistical analyses were performed using Statistical Product and Service Solutions (SPSS) version 23.0 (SPSS Inc., Chicago, IL, USA) and R software (version 4.4.1). The normality of continuous variables was assessed via the Kolmogorov-Smirnov one-sample test. Non-normally distributed continuous variables are reported as median (interquartile range, IQR), and normally distributed variables as mean (standard deviation, SD). Categorical variables were expressed as frequencies and percentages. Independent samples t-tests, Mann-Whitney U tests, and chi-square tests were used as appropriate to compare socio-demographic and clinical characteristics between patients and HCs, as well as between patients with and without SI. Analysis of covariance (ANCOVA) was used to compare FBG levels between patients and HCs, controlling for demographic and clinical variables that differed significantly between the two groups. A Firth penalized logistic regression model was employed to identify factors independently associated with SI among patients. SI was defined as the dependent variable, and independent variables were determined *a priori* based on clinical expertise, including age, sex, relationship with family, fluoxetine equivalents, modified HAMD-24 total score, TSH, and FBG. Model stability was assessed via 1000 bootstrap resamples. A sensitivity analysis was performed to verify the robustness of the results by comparing regression models with the modified HAMD-24 total score versus those with the HAMD-24 total score. Subsequently, receiver operating characteristic (ROC) curve analysis was performed as an exploratory analysis to calculate the area under the curve (AUC) and assess the within-sample discrimination of these independent factors for SI. Spearman’s rank correlation analysis was employed to evaluate the correlations between the severity of SI and other clinical variables. Multivariate linear regression models (Forward: LR) were further applied to verify any significant correlations (*P* < 0.05) identified in the correlation analyses. A two-tailed *P* value < 0.05 was considered statistically significant for all analyses.

## 3 Results

### 3.1 Comparisons between patients and HCs

**Figure 1:**
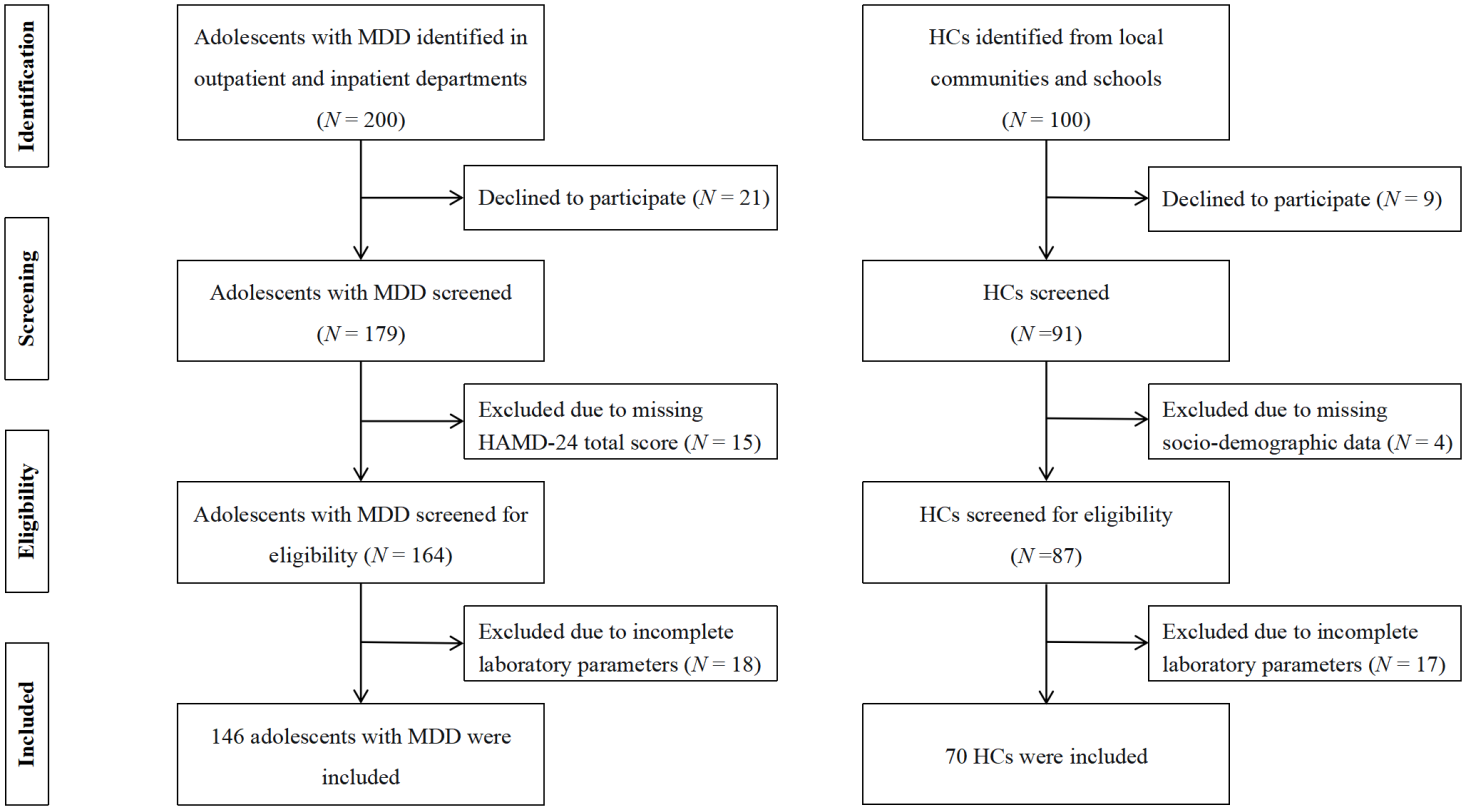
Flow diagram of participant recruitment. (MDD, major depressive disorder; HCs, healthy controls; HAMD-24, 24-item Hamilton depression rating scale).

As shown in **Figure 1**, a total of 146 patients (83 from the Fourth Affiliated Hospital of Anhui Medical University and 63 from the Fourth People’s Hospital of Hefei) and 70 HCs met the eligibility criteria and were enrolled in the analyses. Of these patients, 79 were recruited from outpatient departments and 67 from inpatient departments. As all missing data arose during participant screening and no missing values were present in the final analytical sample, data imputation was not conducted. As shown in **Table 1**, the patients had a lower proportion of males, a lower proportion of good relationship with family, and lower FBG levels than HCs (all *P* < 0.05). ANCOVA showed that the difference in FBG levels (*F* (1, 212) = 12.280, *P =* 0.001) between the two groups remained significant after controlling for sex and relationship with family. There were no statistically significant differences in TSH and lipid profile levels between the patients and HCs (all *P* > 0.05).

**Table 1:** Socio-demographic and clinical characteristics of patients and healthy controls.

Variables	Patients	Healthy controls	Statistics	
	(*N* = 146)	(*N* = 70)	*χ^2^*	*P*
	*N* (%)	*N* (%)		
Male	41 (28.1)	42 (60.0)	20.372^a^	**< 0.001**
Parental marital status			3.581^a^	0.058
Normal	106 (72.6)	59 (84.3)		
Separation, divorce or others	40 (27.4)	11 (15.7)		
Relationship with family			46.058^a^	**< 0.001**
Good	43 (29.5)	55 (78.6)		
Fair/poor	103 (70.5)	15 (21.4)		
History of suicide attempts	96 (65.8)	–		
Antidepressants				
None	46 (31.5)	–		
SSRIs	90 (61.6)	–		
Others	10 (6.8)	–		
Mood stabilizers	15 (10.3)	–		
Atypical antipsychotics	14 (9.6)	–		
	Median (Q_1_, Q_3_)	Median (Q_1_, Q_3_)	*Z*	*P*
Age (years)	15.00 (14.00, 17.00)	15.00 (14.00, 16.00)	-1.696^b^	0.090
BMI (kg/m^2^)	20.23 (18.25, 22.36)	20.68 (18.35, 22.97)	-0.701^b^	0.483
Age of onset (years)	14.00 (13.00, 15.00)	–		
Duration of illness (months)	12.50 (10.00, 36.00)	–		
Fluoxetine equivalents (mg/d)	20.00 (0, 40.00)	–		
HAMD-24 total score	49.00 (40.00, 59.00)	–		
PANSI total score	30.00 (22.00, 35.00)	–		
Laboratory parameters				
TSH (μIU/mL)	1.72 (1.22, 2.56)	2.14 (1.28, 2.70)	-1.582^b^	0.114
FBG (mmol/L)	5.00 (4.50, 5.43)	5.30 (5.10, 5.70)	-4.559^b^	**< 0.001**
TC (mmol/L)	3.77 (3.18, 4.35)	3.72 (3.40, 4.15)	-0.409^b^	0.682
TG (mmol/L)	0.98 (0.75, 1.52)	0.90 (0.75, 1.19)	-0.971^b^	0.331
HDL-C (mmol/L)	1.13 (0.96, 1.35)	1.25 (0.99, 1.45)	-1.911^b^	0.056
LDL-C (mmol/L)	1.93 (1.46, 2.44)	1.93 (1.53, 2.35)	-0.229^b^	0.819

SSRIs, selective serotonin reuptake inhibitors; BMI, body mass index; HAMD-24, 24-item Hamilton Depression Rating Scale; PANSI, Positive and Negative Suicide Ideation Inventory; TSH, thyroid-stimulating hormone; FBG, fasting blood glucose; TC, total cholesterol; TG, triglyceride; HDL-C, high-density lipoprotein cholesterol; LDL-C, low-density lipoprotein cholesterol; a, chi-square test; b, Mann-Whitney U test; Bolded *P* values < 0.05.

### 3.2 Comparisons between patients with and without SI

**Figure 2:**
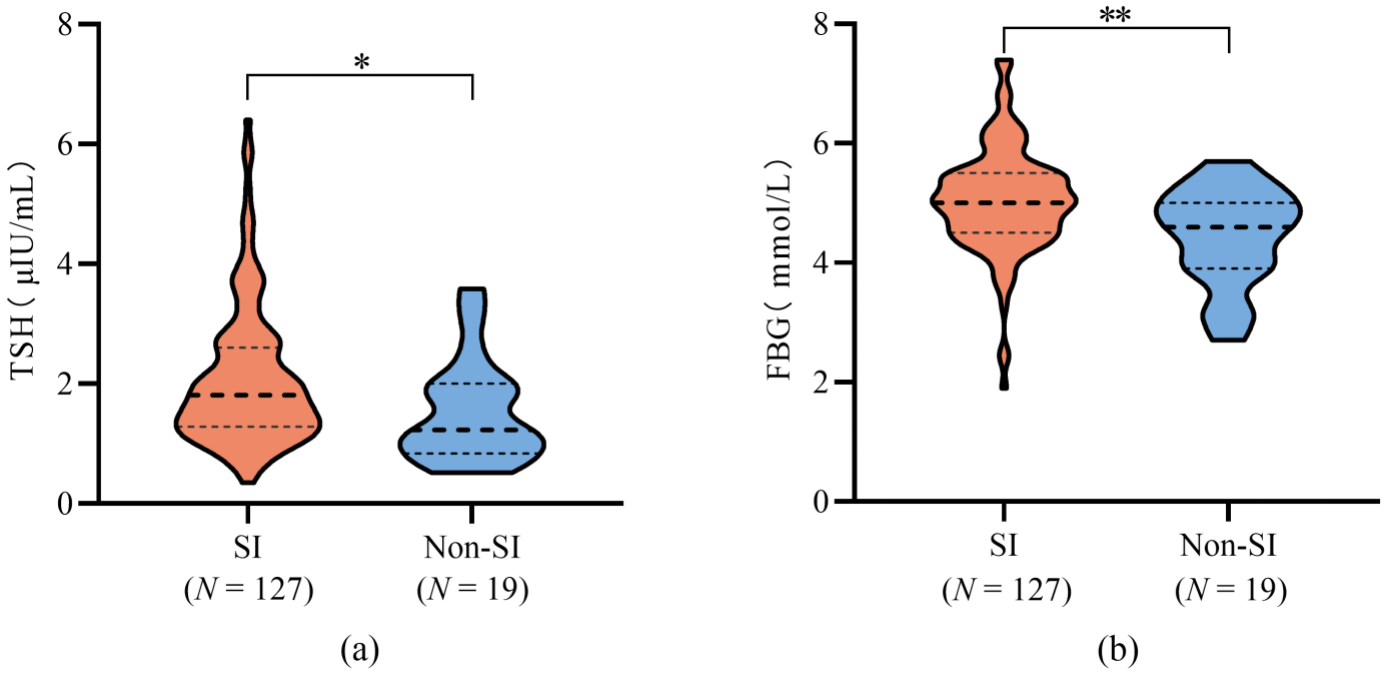
Comparison of TSH **(a)** and FBG **(b)** levels between patients with and without suicidal ideation. (TSH, thyroid-stimulating hormone; FBG, fasting blood glucose; SI, suicidal ideation; *P < 0.05, **P < 0.01.).

As shown in **Table 2**, patients with SI had a lower proportion of good relationship with family, a higher proportion of history of suicide attempts, a longer duration of illness, and higher total scores of modified HAMD-24 and PANSI than those without SI (all *P* < 0.05). In terms of laboratory parameters, patients with SI had higher levels of TSH and FBG than those without SI (all *P* < 0.05) (**Figure 2**).

**Table 2:** Socio-demographic and clinical characteristics of patients with and without suicidal ideation.

Variables	SI	Non-SI	Statistics	
	(*N* = 127)	(*N* = 19)	*χ^2^*	*P*
	*N* (%)	*N* (%)		
Male	33 (26.0)	8 (42.1)	2.127^a^	0.145
Parental marital status			1.480^a^	0.224
Normal	90 (70.9)	16 (84.2)		
Separation, divorce or others	37 (29.1)	3 (15.8)		
Relationship with family			11.943^a^	**0.001**
Good	31 (24.4)	12 (63.2)		
Fair/poor	96 (75.6)	7 (36.8)		
History of suicide attempts	91 (71.7)	5 (26.3)	15.087^a^	**< 0.001**
Antidepressants			3.528^a^	0.171
None	37 (29.1)	9 (47.4)		
SSRIs	82 (64.6)	8 (42.1)		
Others	8 (6.3)	2 (10.5)		
Mood stabilizers	14 (11.0)	1 (5.3)	0.595^b^	0.693
Atypical antipsychotics	13 (10.2)	1 (5.3)	0.471^b^	0.695
Abnormal TSH	4 (3.1)	0	0.615^b^	0.433
Abnormal FBG	19 (15.0)	4 (21.1)	0.462^b^	0.497
	Median (Q_1_, Q_3_)	Median (Q_1_, Q_3_)	*t/Z*	*P*
Age (years)	15.00 (14.00, 17.00)	15.00 (15.00, 16.00)	0.073^c^	0.942
BMI (kg/m^2^)	20.20 (18.24, 22.09)	20.99 (18.25, 24.27)	-1.131^c^	0.258
Age of onset (years)	13.00 (12.00, 15.00)	14.00 (13.00, 16.00)	-1.760^c^	0.078
Duration of illness (months)	18.00 (11.00, 36.00)	12.00 (4.00, 12.00)	-2.438^c^	**0.015**
Fluoxetine equivalents (mg/d)	20.00 (0, 40.00)	10.00 (0, 20.00)	-1.863^c^	0.062
Modified HAMD-24 total score, mean (SD)	27.47 (7.45)	19.11 (8.06)	-4.405^d^	**< 0.001**
PANSI total score	53.00 (45.00, 61.00)	29.00 (23.00, 33.00)	-6.843^c^	**< 0.001**
Laboratory parameters				
TSH (μIU/mL)	1.81 (1.28, 2.60)	1.23 (0.84, 2.00)	-2.289^c^	**0.022**
FBG (mmol/L)	5.00 (4.50, 5.50)	4.60 (3.90, 5.00)	-2.705^c^	**0.007**
TC (mmol/L)	3.77 (3.25, 4.38)	3.30 (2.62, 4.53)	-1.062^c^	0.288
TG (mmol/L)	0.98 (0.74, 1.48)	1.05 (0.77, 1.66)	-0.762^c^	0.446
HDL-C (mmol/L)	1.13 (0.97, 1.34)	1.08 (0.77, 1.51)	-0.596^c^	0.551
LDL-C (mmol/L)	1.97 (1.55, 2.43)	1.57 (1.05, 2.66)	-1.408^c^	0.159

SI, suicidal ideation; SSRIs, selective serotonin reuptake inhibitors; BMI, body mass index; HAMD-24, 24-item Hamilton Depression Rating Scale; PANSI, Positive and Negative Suicide Ideation Inventory; TSH, thyroid-stimulating hormone; FBG, fasting blood glucose; TC, total cholesterol; TG, triglyceride; HDL-C, high-density lipoprotein cholesterol; LDL-C, low-density lipoprotein cholesterol; SD, standard deviation; a, chi-square test; b, Fisher’s exact test; c, Mann-Whitney U test; d, independent samples t-test. Abnormal TSH was defined as TSH levels outside the reference range (0.35-4.94 μIU/mL). Abnormal FBG was defined as FBG levels outside the reference range (3.9-6.1 mmol/L). Bolded *P* values < 0.05.

### 3.3 Independent factors associated with SI in patients

As shown in **Table 3**, Firth penalized logistic regression analyses revealed that a worse relationship with family [odds ratio (OR) = 4.103, 95% confidence interval (CI): 1.284-14.433], a higher total score of modified HAMD-24 (OR = 1.121, 95% CI: 1.032-1.230), and higher levels of TSH (OR = 2.145, 95% CI: 1.083-5.067) and FBG (OR = 1.865, 95% CI: 1.073-3.413) were independently associated with a higher risk of SI in adolescents with MDD (all *P* < 0.05). Bootstrap resampling with 1,000 replicates revealed robust positive associations of relationship with family, modified HAMD-24 total score, and levels of TSH and FBG with SI, and their 95% bootstrap CIs all excluded zero. Sensitivity analysis demonstrated that the ORs and 95% CIs for the independent variables were not appreciably different between models with and without the suicide item, supporting the robustness and reliability of the findings (**Supplementary Table 1**).

**Table 3:** Independent factors associated with suicidal ideation in patients.

Variables	*P*	OR	95% CI	
			Lower	Upper
Age	0.736	1.076	0.701	1.667
Sex (ref. female)				
Male	0.593	0.724	0.224	2.447
Relationship with family (ref. good)				
Fair/poor	**0.017**	4.103	1.284	14.433
Fluoxetine equivalents (mg/d)	0.068	1.027	0.998	1.063
Modified HAMD-24 total score	**0.006**	1.121	1.032	1.230
TSH (μIU/mL)	**0.026**	2.145	1.083	5.067
FBG (mmol/L)	**0.027**	1.865	1.073	3.413

HAMD-24, 24-item Hamilton Depression Rating Scale; TSH, thyroid-stimulating hormone; FBG, fasting blood glucose; ref., reference; OR, odds ratio; CI, confidence interval; Bolded *P* values < 0.05.

### 3.4 ROC curve analyses of SI in patients

**Figure 3:**
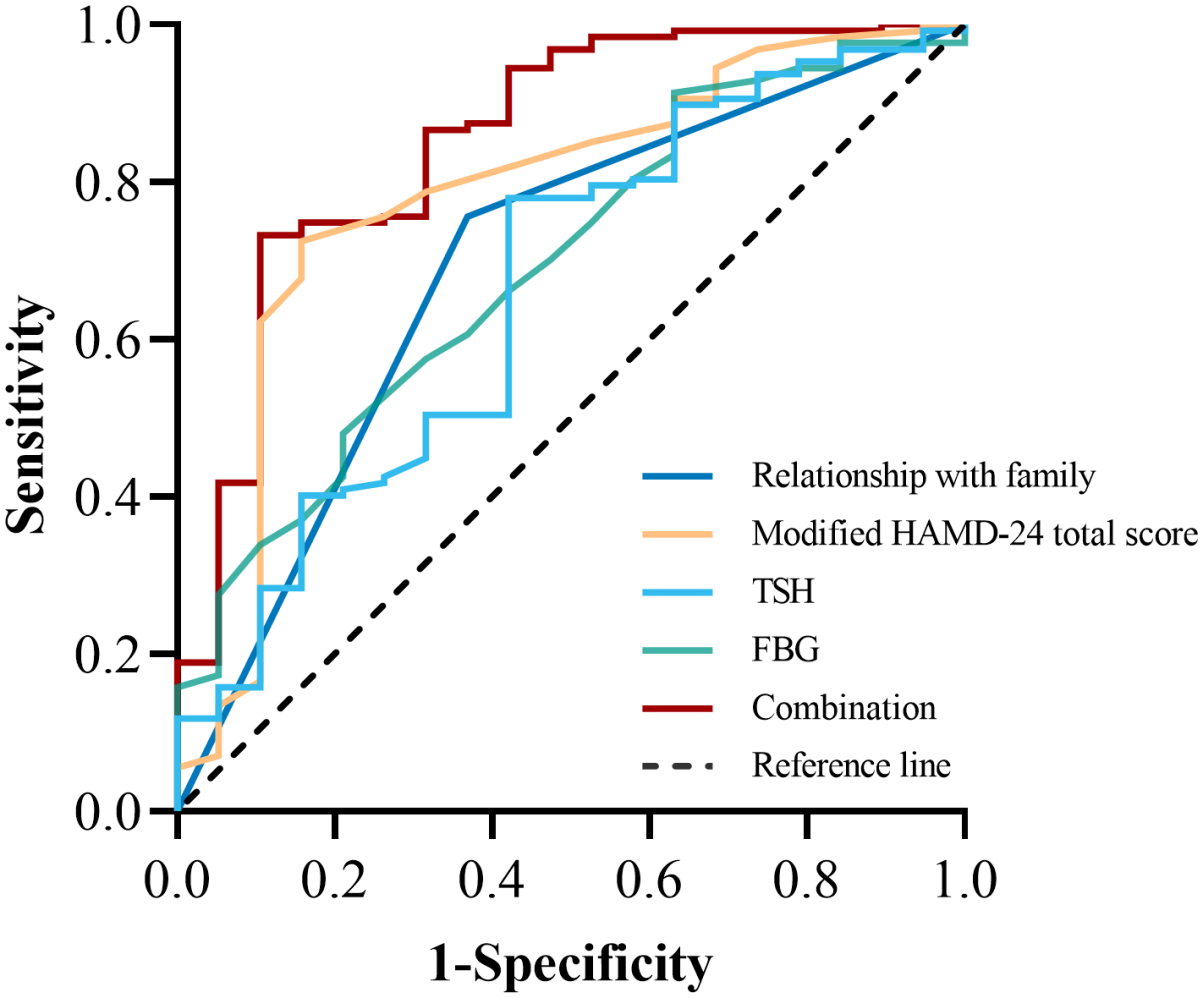
ROC curves of relationship with family, modified HAMD-24 total score, TSH levels, FBG levels, and their combination for discriminating concurrent suicidal ideation in patients. (ROC, receiver operating characteristic; HAMD-24, 24-item Hamilton depression rating scale; TSH, thyroid-stimulating hormone; FBG, fasting blood glucose).

ROC curve analysis was further conducted to assess the exploratory within-sample discrimination of the significant variables from the logistic regression model for SI. As shown in **Table 4** and **Figure 3**, the AUC for relationship with family was 0.694 (95% CI: 0.560-0.828), for modified HAMD-24 total score was 0.785 (95% CI: 0.664-0.905), for TSH levels was 0.663 (95% CI: 0.526-0.800), for FBG levels was 0.692 (95% CI: 0.570-0.815), and for the combination of the four indicators was 0.851 (95% CI: 0.750-0.952), with a sensitivity of 73.2% and a specificity of 89.5%. Compared with any single variable, the combination of the four variables achieved a larger AUC and higher specificity, reflecting a better ability to discriminate SI and a correspondingly lower false-positive rate.

**Table 4:** ROC curve analysis of suicidal ideation in patients.

Variables	AUC	95% CI	*P*	Sensitivity (%)	Specificity (%)
Relationship with family	0.694	0.560~0.828	**0.007**	75.6	63.2
Modified HAMD-24 total score	0.785	0.664~0.905	**< 0.001**	72.4	84.2
TSH (μIU/mL)	0.663	0.526~0.800	**0.022**	78.0	57.9
FBG (mmol/L)	0.692	0.570~0.815	**0.007**	91.3	36.8
Combination	0.851	0.750~0.952	**< 0.001**	73.2	89.5

HAMD-24, 24-item Hamilton Depression Rating Scale; TSH, thyroid-stimulating hormone; FBG, fasting blood glucose; AUC, area under the curve; CI, confidence interval; Bolded *P* value < 0.05.

### 3.5 Independent correlates of the severity of SI in patients

As shown in **Supplementary Table 2**, several socio-demographic and clinical variables were significantly correlated with the severity of SI in patients. Model diagnostics were conducted to evaluate the assumptions of linear regression. Examination of partial regression plots confirmed the linear relationships between variables. All variance inflation factor values were below 5, indicating the absence of multicollinearity. The maximum Cook’s distance was below 1, suggesting that no influential observations were present. Further multivariate linear regression analysis revealed that the severity of SI was negatively associated with age of onset (*β* = -0.174, *P* = 0.009), and was positively associated with worse relationship with family (*β* = 0.192, *P* = 0.007), history of suicide attempts (*β* = 0.215, *P* = 0.003), modified HAMD-24 total score (*β* = 0.316, *P* < 0.001), and TSH levels (*β* = 0.175, *P* = 0.009) (**Supplementary Table 3**).

## 4 Discussion

In this study, we examined the levels of TSH and glycolipid metabolism parameters, as well as their associations with SI in adolescents with MDD. Unlike previous findings in adults with MDD (39), we found that adolescents with MDD had lower FBG levels than HCs. One possible explanation is that adolescents with MDD frequently experience appetite loss and somatic symptoms, which in turn lead to being underweight and lower FBG levels (29, 40). Furthermore, Yao et al. proposed that SSRIs could improve insulin sensitivity and glycemic control, but combination therapy may carry a risk of hypoglycemia (41). In addition, there was no significant difference in TSH levels between adolescents with MDD and HCs in this study. Similarly, a nationwide prospective study from Germany and a Mendelian randomization study demonstrated that TSH and free thyroxine levels within the normal range were not significantly associated with MDD (42, 43). These findings suggest that minor variations in thyroid function may not be significantly causally associated with the risk of MDD. Given the substantial heterogeneity between patients and HCs, the comparison between the two groups should be considered a secondary finding. This study also found that the prevalence of SI among adolescents with MDD was 87.0%, which is significantly higher than the 14.3%-22.6% reported in the general adolescent population (44). Therefore, the high prevalence of SI in adolescents with MDD underscores the urgent need to identify its associated factors.

In this study, we also found that relationship with family and depressive symptoms were independently associated with concurrent SI in adolescents with MDD. First, adolescents with MDD who had poorer family relationships were more likely to experience SI. Previous studies have indicated that adolescents with MDD who have worse family relationships are more prone to feelings of isolation and helplessness when encountering setbacks, which may lead them to adopt negative coping strategies such as self-harm and suicidal behavior (45). Conversely, a meta-analysis revealed that family therapy can significantly reduce SI in adolescents with MDD by modifying interaction patterns among family members and improving overall family functioning (46). Second, this study found that patients with more severe depressive symptoms were more likely to have concurrent SI. A recent randomized, double-blind clinical trial also revealed that depressive symptoms were an important predictor of SI, and that early standardized antidepressant treatment was key to reducing the risk of SI in patients with MDD (47).

In addition, adolescents with MDD who had a history of suicide attempts exhibited higher levels of SI. Suicidal behavior often recurs throughout the lifespan, and a meta-analysis showed that adolescents with a history of suicide attempts may face an elevated risk of suicidality in young adulthood (48). Moreover, studies by DeVille et al. (49) and Qin et al. (50) demonstrated that patients with a history of suicide attempts exhibited heightened pain tolerance, along with more severe depressive symptoms and perceived stress, which may collectively exacerbate the severity of SI. With regard to disease characteristics, age of onset was negatively associated with the severity of SI among adolescents with MDD. Similarly, a multicenter study enrolling patients with MDD reported that the severity of SI was negatively correlated with age of onset (51). This may be explained by the fact that patients with an earlier onset experience negative emotions such as pessimism and hopelessness for a longer duration, which in turn leads to more severe SI.

In terms of laboratory parameters, serum TSH levels were significantly associated with the occurrence and severity of SI in adolescents with MDD, consistent with previous findings in adult populations (52). However, a meta-analysis involving 2807 participants indicated that TSH levels were negatively associated with the risk of recent SI, yet positively associated with a history of suicide attempts (53). Two other studies conducted in adults and adolescents with MDD reported no significant association between suicidal behavior and TSH levels (54, 55). The dynamic and complex involvement of TSH in suicidal behavior is reflected by the inconsistent results reported across these studies. Currently, there is limited research on the mechanisms underlying the association between thyroid function alterations and suicidal behavior. It has been proposed that the chronic stress experienced by individuals who exhibit suicidal behavior may provoke immune dysregulation, which in turn contributes to changes in thyroid function (56, 57). In addition, the association between TSH levels and SI may be mediated by alterations in brain function. For instance, a neuroimaging study demonstrated that patients with SI had elevated TSH levels, which were associated with decreased dynamic amplitude of low-frequency fluctuations in the right anterior cingulate cortex (58).

Notably, elevated FBG levels were also independently associated with the occurrence of SI, but showed no significant correlation with SI severity. This discrepancy may suggest a threshold effect, whereby elevated FBG levels act as a trigger for the occurrence of SI but do not linearly drive its severity. The severity of SI may be primarily determined by more complex socio-demographic and biological factors, with the role of FBG being diminished. Previous studies have confirmed that patients with MDD often exhibit varying degrees of glucose metabolism alterations, including abnormalities in levels of FBG, insulin, and glucagon (59, 60). Further studies showed that patients with MDD who also had glucose disturbances had a 1.88-fold higher risk of suicide compared to those without such disturbances (61). Early studies indicated that abnormal blood glucose levels could impair patients’ self-control, which might in turn increase the risk of SI (62). According to forensic pathology evidence, deranged glucose metabolism might serve as a prodromal biological indicator or a concomitant symptom of suicidal behavior (63). Additionally, patients with suicidal behavior might exhibit hyperactivation of the hypothalamic-pituitary-adrenal axis, which could promote glucocorticoid release and elevated FBG levels (64, 65). Nevertheless, given the cross-sectional nature of this study, these hypothetical mechanisms need to be further validated in follow-up studies. In contrast to previous findings (66, 67), the present study did not identify significant associations between SI and lipid profiles. This discrepancy may be attributable to differences in study population characteristics, disease status, and the assessment tools used.

Several limitations of this study should be acknowledged. First, the cross-sectional design precludes any causal interpretation of the observed associations between alterations in TSH and FBG levels and the risk of SI. In addition, the distributions of TSH and FBG showed considerable overlap between the SI and non-SI groups, which may limit their value for individual risk stratification. Second, patients were recruited from two hospitals in Anhui Province, resulting in a relatively limited sample size and a homogeneous population. This may have introduced selection bias and reduced statistical power, thereby limiting the generalizability of the findings. Third, potential confounders, including study site, nutritional status, pubertal stage, and physical activity, were not fully adjusted for. Moreover, given that the study period overlapped with the COVID-19 pandemic, inadequate control for this confounder may bias the effect estimates. Thus, the biological findings reported in this study should be explicitly regarded as exploratory. Fourth, although the SI classification criteria have been applied in adolescent and college student samples, they lack specific validation in adolescents with MDD, warranting caution in the interpretation of the associated findings. Future studies could adopt a prospective, multicenter cohort design and incorporate regular follow-up assessments and cross-lagged panel models to verify the causal relationships between variables. Meanwhile, more comprehensive clinical data and biological markers should be included to provide a theoretical basis for the development of targeted intervention strategies.

## 5 Conclusion

In this cross-sectional clinical sample of adolescents with MDD, serum TSH and FBG levels were associated with concurrently assessed SI status in exploratory analyses. These findings require confirmation in larger prospective studies using well-validated SI definitions among adolescents with MDD, more complete assessments of clinical and treatment-related confounders, and appropriate internal and external validation before any clinical application can be considered.

## Data Availability

The original contributions presented in the study are included in the article/**Supplementary Material**. Further inquiries can be directed to the corresponding authors.
