# Supplementary material for: Thyroid-stimulating hormone, fasting blood glucose and suicidal ideation in Chinese adolescents with major depressive disorder: a cross-sectional study
Source: Front Psychiatry. 2026 Jul 20;17:1892174. doi: 10.3389/fpsyt.2026.1892174 (PMC13429666; doi:10.3389/fpsyt.2026.1892174)
Supplement: Supplementary file 1 [file Table1.docx]

**Supplementary Appendix**

**Table of Contents**

**Supplementary Table S1** Independent factors associated with suicidal ideation in patients (based on the HAMD total score).

**Supplementary Table S2** Correlations of socio-demographic and clinical variables with severity of suicidal ideation in patients.

**Supplementary Table S3** Independent correlates of severity of suicidal ideation in patients.

**Supplementary Table S1** Independent factors associated with suicidal ideation in patients (based on the HAMD total score).

| **Variables** | ***P*** | **OR** | **95% CI** | |
| --- | --- | --- | --- | --- |
|  |  |  | **Lower** | **Upper** |
| Relationship with family (ref. good) |  |  |  |  |
| Fair/poor | **0.032** | 3.607 | 1.115 | 12.571 |
| HAMD-24 total score | **0.002** | 1.113 | 1.040 | 1.203 |
| TSH (μIU/mL) | **0.042** | 2.014 | 1.022 | 4.718 |
| FBG (mmol/L) | **0.034** | 1.889 | 1.048 | 3.651 |
| HAMD-24, 24-item Hamilton Depression Rating Scale; TSH, thyroid-stimulating hormone; FBG, fasting blood glucose; ref., reference; OR, odds ratio; CI, confidence interval; Bolded *P* values < 0.05. | | | | |

**Supplementary Table S2** Correlations of socio-demographic and clinical variables with severity of suicidal ideation in patients.

| **Variables** | ***r***^a^ | **P** |
| --- | --- | --- |
| General socio-demographic and clinical data |  |  |
| Age (years) | -0.291 | **< 0.001** |
| BMI (kg/m^2^) | -0.007 | 0.934 |
| Sex (male/female) | -0.272 | **0.001** |
| Parental marital status (normal/separation, divorce or others) | 0.153 | 0.065 |
| Relationship with family (good/fair/poor) | 0.356 | **< 0.001** |
| History of suicide attempts (yes/no) | 0.407 | **<0.001** |
| Antidepressants (none/SSRIs/others) | -0.004 | 0.962 |
| Mood stabilizers | 0.005 | 0.954 |
| Atypical antipsychotics | 0.009 | 0.913 |
| Abnormal TSH | 0.130 | 0.118 |
| Abnormal FBG | -0.078 | 0.351 |
| Age of onset (years) | -0.326 | **< 0.001** |
| Duration of illness (months) | 0.135 | 0.105 |
| Fluoxetine equivalents (mg/d) | 0.075 | 0.371 |
| Modified HAMD-24 total score | 0.479 | **< 0.001** |
| Laboratory parameters |  |  |
| TSH (μIU/mL) | 0.249 | **0.002** |
| FBG (mmol/L) | 0.121 | 0.146 |
| TC (mmol/L) | 0.027 | 0.747 |
| TG (mmol/L) | 0.069 | 0.405 |
| HDL-C (mmol/L) | -0.064 | 0.442 |
| LDL-C (mmol/L) | 0.062 | 0.459 |
| BMI, body mass index; SSRIs, selective serotonin reuptake inhibitors; HAMD-24, 24-item Hamilton Depression Rating Scale; TSH, thyroid-stimulating hormone; FBG, fasting blood glucose; TC, total cholesterol; TG, triglyceride; HDL-C, high-density lipoprotein cholesterol; LDL-C, low-density lipoprotein cholesterol; a, spearman’s rank correlation. Abnormal TSH was defined as TSH levels outside the reference range (0.35-4.94 μIU/mL). Abnormal FBG was defined as FBG levels outside the reference range (3.9-6.1 mmol/L). Bolded *P* values < 0.05. | | |

**Supplementary Table S3** Independent correlates of severity of suicidal ideation in patients.

| **Variables** | **B** | **SE** | ***β*** | ***P*** |
| --- | --- | --- | --- | --- |
| Relationship with family (ref. good) |  |  |  |  |
| Fair/poor | 5.349 | 1.937 | 0.192 | **0.007** |
| History of suicide attempts (ref. no) |  |  |  |  |
| Yes | 5.749 | 1.879 | 0.215 | **0.003** |
| Age of onset (years) | -1.155 | 0.439 | -0.174 | **0.009** |
| Modified HAMD-24 total score | 0.503 | 0.117 | 0.316 | **< 0.001** |
| TSH (μIU/mL) | 1.967 | 0.741 | 0.175 | **0.009** |
| *F* | 20.308 | | | |
| *R*^2^/adjusted *R*^2^ | 0.420/0.400 | | | |
| HAMD-24, 24-item Hamilton Depression Rating Scale; TSH, thyroid-stimulating hormone; ref., reference; SE, standard error. Bolded *P* values < 0.05. | | | | |
