# Supplementary material for: Thyroid-stimulating hormone, fasting blood glucose and suicidal ideation in Chinese adolescents with major depressive disorder: a cross-sectional study
Source: Front Psychiatry. 2026 Jul 20;17:1892174. doi: 10.3389/fpsyt.2026.1892174 (PMC13429666; doi:10.3389/fpsyt.2026.1892174)
Supplement: Supplementary file 2 [file Table2.docx]

STROBE Statement—Checklist of items that should be included in reports of ***cross-sectional studies***

|  | Item No | Recommendation | Response |
| --- | --- | --- | --- |
| **Title and abstract** | 1 | (*a*) Indicate the study’s design with a commonly used term in the title or the abstract | The title clearly states this is a cross-sectional study; the abstract also describes the cross-sectional design. |
|  |  | (*b*) Provide in the abstract an informative and balanced summary of what was done and what was found | The abstract includes background, methods, key results (e.g., findings from regression analyses and ROC curve analysis), and conclusions. |
| Introduction | | |  |
| Background/rationale | 2 | Explain the scientific background and rationale for the investigation being reported | The first four paragraphs of the introduction provide background on MDD, suicide, TSH, and glycolipid metabolism. |
| Objectives | 3 | State specific objectives, including any prespecified hypotheses | The last paragraph of the introduction outlines two primary objectives. |
| Methods | | |  |
| Study design | 4 | Present key elements of study design early in the paper | Section 2.1 states “cross-sectional study.” |
| Setting | 5 | Describe the setting, locations, and relevant dates, including periods of recruitment, exposure, follow-up, and data collection | • Setting: The Fourth Affiliated Hospital of Anhui Medical University and Hefei Fourth People’s Hospital (Anhui Province, China).  • Recruitment period: October 2020 to March 2022. No follow-up was performed for this cross-sectional study. |
| Participants | 6 | (*a*) Give the eligibility criteria, and the sources and methods of selection of participants | • Detailed inclusion and exclusion criteria for MDD adolescent patients and HCs.  • Participants were recruited from two hospitals, local communities and schools. |
| Variables | 7 | Clearly define all outcomes, exposures, predictors, potential confounders, and effect modifiers. Give diagnostic criteria, if applicable | • Outcome: Suicidal ideation (SI), defined by PANSI scoring criteria  • Exposures/predictors: TSH, FBG, lipid profiles, relationship with family, depressive symptoms, medication use, etc.  • Diagnostic criteria: MDD diagnosed via SCID-5. |
| Data sources/ measurement | 8* | For each variable of interest, give sources of data and details of methods of assessment (measurement). Describe comparability of assessment methods if there is more than one group | • Socio-demographic and clinical data: Pre-designed questionnaire.  • Depressive symptoms: HAMD-24; SI: PANSI.  • Laboratory indicators: Uniform blood collection, testing instruments, operating procedures and quality control standards for all participants. |
| Bias | 9 | Describe any efforts to address potential sources of bias | • Unified diagnostic tools and standardized assessment procedures.  • Strict inclusion/exclusion criteria to exclude interfering diseases and medications.  • Mentioned exclusion of HAMD item 3 to avoid confounding. |
| Study size | 10 | Explain how the study size was arrived at | Sample size was calculated via G*Power 3.1.9.7 (effect size = 0.50, *α* = 0.05, power = 0.80); Minimum required sample size = 134, final enrolled 146 MDD adolescents. |
| Quantitative variables | 11 | Explain how quantitative variables were handled in the analyses. If applicable, describe which groupings were chosen and why | • Normality tested by Kolmogorov-Smirnov test; Non-normal continuous variables: Median (Q1, Q3); Normal variables: Mean (SD).  • Categorical variables: Frequencies and percentages.  • As reported in previous studies, patients with a mean item score for PSI of ≤ 3.333 and a mean item score for NSI of ≥ 1.625 were considered to have SI. |
| Statistical methods | 12 | (*a*) Describe all statistical methods, including those used to control for confounding | Independent samples t-test, Mann-Whitney U test, Chi-square test, Fisher’s exact test, and Spearman correlation. ANCOVA, logistic regression, and multivariate linear regression (for confounding adjustment). |
|  |  | (*b*) Describe any methods used to examine subgroups and interactions | Subgroup analyses: Comparisons between SI vs. non-SI patients; No interaction analyses were performed |
|  |  | (*c*) Explain how missing data were addressed | As all missing data arose during participant screening and no missing values were present in the final analytical sample, data imputation was not conducted. |
|  |  | (*d*) If applicable, describe analytical methods taking account of sampling strategy | Participants were consecutively recruited from two designated hospitals; Standard statistical methods were applied for this sampling strategy. |
|  |  | (*e*) Describe any sensitivity analyses | Sensitivity analysis was conducted by comparing regression models using original HAMD-24 total score vs. modified HAMD-24 total score to verify result robustness. |
| Results | | |  |
| Participants | 13* | (a) Report numbers of individuals at each stage of study—eg numbers potentially eligible, examined for eligibility, confirmed eligible, included in the study, completing follow-up, and analysed | Total enrolled: 146 MDD adolescents and 70 HCs; Numbers of outpatient/inpatient patients were reported; All eligible participants completed assessments and were included in final analysis (see Figure 1). |
|  |  | (b) Give reasons for non-participation at each stage | Participants who failed to meet inclusion criteria or met exclusion criteria were excluded during screening; Specific exclusion reasons followed the pre-set exclusion standards (see Figure 1). |
|  |  | (c) Consider use of a flow diagram | A participant recruitment flow diagram (see Figure 1) was provided to show the whole screening and enrollment process. |
| Descriptive data | 14* | (a) Give characteristics of study participants (eg demographic, clinical, social) and information on exposures and potential confounders | Socio-demographic, clinical characteristics, medication and laboratory characteristics of patients and HCs, SI/non-SI subgroups were fully presented in Table 1 and Table 2. |
|  |  | (b) Indicate number of participants with missing data for each variable of interest | Clearly stated that no missing data existed in the final analytical dataset. |
| Outcome data | 15* | Report numbers of outcome events or summary measures | Reported the number (*N* = 127) and proportion (87.0%) of participants with SI; Summary statistics (median, mean, proportion) for all outcome and exposure variables were listed in tables. |
| Main results | 16 | (*a*) Give unadjusted estimates and, if applicable, confounder-adjusted estimates and their precision (eg, 95% confidence interval). Make clear which confounders were adjusted for and why they were included | • Unadjusted group comparison results: *P* values, median/mean, proportions.  • Adjusted results: ANCOVA adjusted for sex and relationship with family; Logistic regression reported OR and 95% CI for independent influencing factors. |
|  |  | (*b*) Report category boundaries when continuous variables were categorized | • SI classification based on PANSI cutoffs.  • Categorization boundaries for abnormal TSH (0.35–4.94 μIU/mL) and abnormal FBG (3.9–6.1 mmol/L) were clearly defined. |
|  |  | (*c*) If relevant, consider translating estimates of relative risk into absolute risk for a meaningful time period | Not applicable (OR reported). |
| Other analyses | 17 | Report other analyses done—eg analyses of subgroups and interactions, and sensitivity analyses | Supplementary Tables S2-S3 (correlation, linear regression). Sensitivity analysis reported. |
| Discussion | | |  |
| Key results | 18 | Summarise key results with reference to study objectives | Summarized core findings in accordance with the two research objectives; Compared results with previous literature. |
| Limitations | 19 | Discuss limitations of the study, taking into account sources of potential bias or imprecision. Discuss both direction and magnitude of any potential bias | Discussed limitations including cross-sectional design (no causal inference), single-region recruitment (selection bias), unmeasured potential confounding factors, and limited clinical utility of indicators. |
| Interpretation | 20 | Give a cautious overall interpretation of results considering objectives, limitations, multiplicity of analyses, results from similar studies, and other relevant evidence | Conclusion: These findings require confirmation in larger prospective studies using well-validated SI definitions, more complete assessment of clinical and treatment-related confounders, and appropriate internal and external validation before any clinical application can be considered. |
| Generalisability | 21 | Discuss the generalisability (external validity) of the study results | Indicated that participants were recruited from two local hospitals in Anhui Province; The sample homogeneity may restrict the generalisability of findings to other regions/populations. |
| Other information | | |  |
| Funding | 22 | Give the source of funding and the role of the funders for the present study and, if applicable, for the original study on which the present article is based | All funding projects were listed clearly; Funders had no role in study design, data collection, analysis and manuscript writing. |

*Give information separately for exposed and unexposed groups.

**Note:** An Explanation and Elaboration article discusses each checklist item and gives methodological background and published examples of transparent reporting. The STROBE checklist is best used in conjunction with this article (freely available on the Web sites of PLoS Medicine at http://www.plosmedicine.org/, Annals of Internal Medicine at http://www.annals.org/, and Epidemiology at http://www.epidem.com/). Information on the STROBE Initiative is available at www.strobe-statement.org.
